# Integrative metabolome and transcriptome analyses reveal the coloration mechanism in *Camellia oleifera* petals with different color

**DOI:** 10.1186/s12870-023-04699-6

**Published:** 2024-01-02

**Authors:** Hai-Tao Zeng, Tao Zheng, Qi Tang, Hao Xu, Mengjiao Chen

**Affiliations:** College of Biology Science and Engineering, Qinba Mountain Area Collaborative Innovation Center of Bioresources Comprehensive Development, Qinba State Key Laboratory of Biological Resources and Ecological Environment (Incubation), Shaanxi University of Technology, Shaanxi Province Key Laboratory of Bio-Resources, Hanzhong, 723001 Shaanxi China

**Keywords:** *Camellia oleifera* petals, Transcriptome, Metabolome, Anthocyanin, Transcription factor

## Abstract

**Background:**

*Camellia olelfera* petals are colorful, and have high ornamental value. However, the color formation mechanism of* C. olelfera* petals with different color is still unclear. In our study, WGCNA method was applied to integrate metabolites and transcriptomes to investigate the coloration mechanism of four *C. olelfera* cultivars with different petal colors.

**Results:**

Here, a total of 372 flavonoids were identified (including 27 anthocyanins), and 13 anthocyanins were significantly differentially accumulated in* C. olelfera* petals. Among them, cyanidin-3-O-(6''-O-p-Coumaroyl) glucoside was the main color constituent in pink petals, cyanidin-3-O-glucoside, cyanidin-3-O-galactoside, cyanidin-3-O-rutinoside, and cyanidin-3-O-(6''-O-malonyl) glucoside were the main contributors to candy pink petals, and peonidin-3-O-glucoside was the important color substance responsible for the red petals of *C. oleifera*. Furthermore, six structural genes (*Co4CL1*, *CoF3H1*, *CoF3'H*, *CoANS*, *CoUGT75C1-4*, and *CoUGT75C1-5*), three MYBs (*CoMYB1*, *CoMYB4*, and *CoMYB44-3*), three bHLHs (*CobHLH30*, *CobHLH 77*, and *CobHLH 79–1*), and two WRKYs (*CoWRKY7* and *CoWRKY22*) could be identified candidate genes related to anthocyanins biosynthesis and accumulation, and lead to the pink and red phenotypes. The regulatory network of differentially accumulated anthocyanins and the anthocyanins related genes in *C. olelfera* petals were established.

**Conclusions:**

These findings elucidate the molecular basis of the coloration mechanisms of pink and red color in *C. olelfera* petals, and provided valuable target genes for future improvement of petals color in *C. olelfera*.

**Supplementary Information:**

The online version contains supplementary material available at 10.1186/s12870-023-04699-6.

## Background

*Camellia olelfera* AbelC.oleosa (Lour.) Rehd. is the flower of *Camellia oleifera* belonging to the genus Camellia [1, 2]. *C. oleifera* flower is known for its abundant resources, beautiful flower type, long flowering period, and has high ornamental value [3]. In addition, *C. oleifera* petals have strong antioxidant, anticancer, nourishing, lipid-lowering, hypoglycemic, nourishing, detoxification due to their abundance of nutrients and active substances, including phenols, amino acids, and flavonoids [4, 5]. Most of *C. oleifera* petals color is white, and there are few researches on the variations of different petals colors. Therefore, producers and researchers continue to explore the formation mechanism of *C. oleifera* petals color, enrich the petals color, and fully realize the economic value and ornamental value of *C. oleifera* flower resources.

Anthocyanins display an essential role in the coloration of different parts of plants, which are the main source of pigments in flowers, fruits, leaves and seeds of many angiosperms, and especially exhibit a significant role in the formation of petal color [6, 7]. Petal color, an important indicator, reflects the growth status, reproductive capacity and ability to adapt to the environment of plants [8, 9]. Anthocyanin is a water-soluble substance that exists in the cell vacuoles of petals, which could produce white, pink, red, blue, and purple colors [10, 11]. In recent years, many scholars have begun focus on the anthocyanins in different petals and the molecular mechanism of flower color formation [12, 13]. The anthocyanin biosynthesis, accumulation pathway and related gene expression have been extensively investigated in many plants [14, 15]. The biosynthesis pathway of anthocyanin belongs to a specific branch pathway of flavonoid biosynthesis pathway, and the biosynthesis of anthocyanins are regulated by structural genes and transcription factors [16, 17]. The biosynthetic pathway of anthocyanin starts from the phenylalanine metabolic pathway, and the biosynthetic reaction is catalyzed by the structural genes encoding the following enzymes: chalcone synthase (*CHS*), chalcone isomerase (*CHI*), flavonol 3′ -hydroxylase (*F3′H*), flavanone-3′ 5′ -hydroxylase (*F3′5′H*), dihydroflavonol-4-reductase (*DFR*), anthocyanin synthase (*ANS*/*LDOX*), and UDP‐glucose: flavonoid 3‐Oglucosyltransferase (*UFGT*) [18]. Transcription factors also display a vital role in anthocyanin biosynthesis, which indirectly regulate anthocyanin biosynthesis by regulating the transcription and expression of structural genes. And, transcription factor families regulating anthocyanin biosynthesis pathway mainly include MYB, bHLH and WD40 [19, 20]. These transcription factors could form MBW complex and activate the promoter of structural genes, thereby regulating the transcription and expression of anthocyanin structural genes [21, 22].

Petals color diversity is mainly caused by the different types and contents of anthocyanins [23]. The main coloring substance of red purple and red *Rhododendron triflorum* is cyanidin, whereas the main color-producing pigment in *Rhododendron nivale* and *Rhododendron oreotrephes* is malvidin [24]. The cyanidin-3-O- (6’’-O-malonyl) glucoside, and cyanidin-3-O-rutinoside, peonidin-3-O-glucoside, cyanidin-3-O-glucoside, and pelargonidin-3-O-glucoside were the main anthocyanins in *Camellia japonica* petals, which were the main coloring substances responsible *C. japonica* petals [6]. Pink tea tree flower has more anthocyanin content than white tea tree flower, such as cyanidin-O-syringic acid, cyanidin-3-O-glucoside and petunidin-3-O-glucoside pigment [3]. Peonidin derivatives were the main coloring compounds that determined the *Rosa rugosa* petals color, and cyanidin-3,5-O-diglucoside and peonidin 3,5-O-diglucoside are two dominant anthocyanins in *R. rugosa* petals, among which the total content of cyanidin-3,5-O-diglucoside determines the color intensity of the petals, such as pink or purple, light red or deep red [25]. With the development of omics technology, more researches have combined metabolome and transcriptome to explore the traits formation mechanism and regulatory genes of horticultural plants. However, the types and contents of anthocyanins in *C. oleifera* petals have not been reported, and the formation mechanism of petal color is still unclear.

In the present study, UPLC-MS/MS technology was to comprehensively characterize the differentially accumulated anthocyanins compounds in four *C. oleifera* petals with different color, and a comprehensive RNA-seq experiment was to screen out the potential genes related to anthocyanins accumulation. The results of this study were helpful to understand the anthocyanin biosynthesis in pink, candy pink, and red *C. oleifera* petals, and provide valuable insights for further study of the regulatory network of anthocyanin biosynthesis and lay an important theoretical guidance for improvement and breeding specific color of *C. oleifera* petals.

## Materials and methods

### Plant materials

**Fig. 1:**
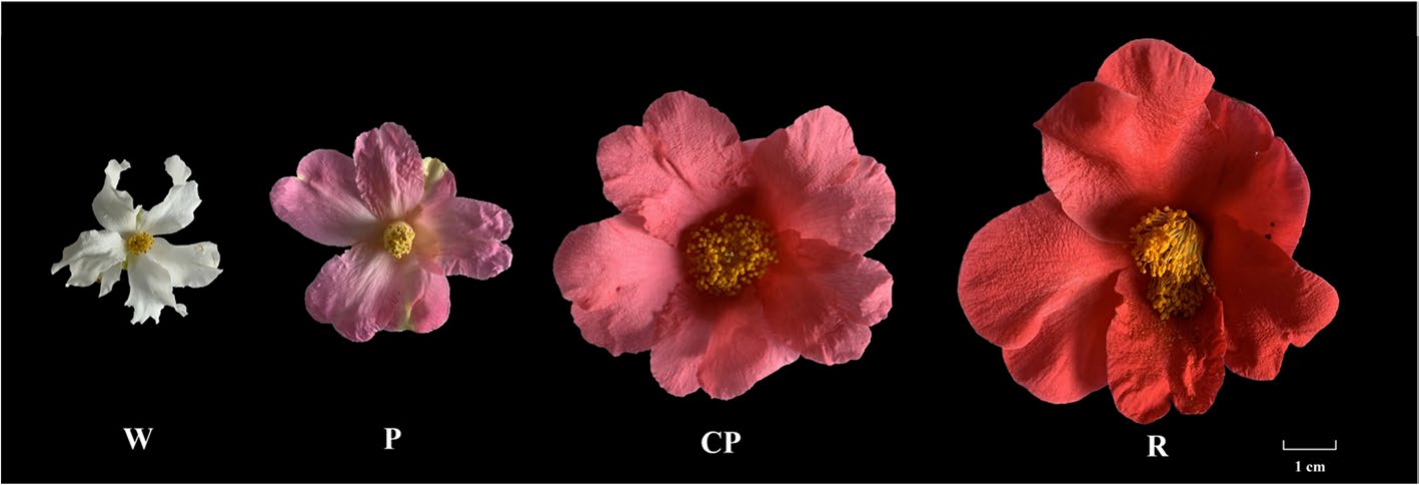
Different flower colors among four *C. oleifera* varieties, namely “*C. yuhsienensis*” (W), “*C. taishunensis*” (P), “*C. reticulate*” (CP), and “*C. chekiangoleosa*” (R)

Four *C. oleifera* cultivars with different petal color, cultivated in Shaanxi *C. oleifera* germplasm resources repository in Nanzheng District, Shaanxi Province, China, with the tree age of 45 years, were selected as the experimental materials. The four *C. oleifera* cultivars, namely “*Camellia yuhsienensis*” (White, W), “*Camellia reticulate*” (Pink, P), “*Camellia semiserrata*” (Candy pink, CP), and “*Camellia chekiangoleosa*” (Red, R), were white, pink, candy pink and red, respectively (Fig. 1). Petals were collected with three biological replicates on March 8, 2023. Then, the petals were immediately stripped, put into liquid nitrogen, and brought back to the laboratory for storage at -80 °C for the anthocyanin determination and RNA extraction.

### Identification and quantitative analysis of metabolites

The abundances of specific anthocyanin compounds were then quantified with ultra performance liquid chromatography (UPLC)-tandem mass spectrometry (MS/MS). Fresh petals samples were freeze-dried, ground to powder in a grinder (MM 400, Retsch) at 30 Hz for 1.5 min, then stored at -80 °C until further analysis. For each sample, 100 mg of powder was extracted in 1.0 mL of methanol. The extracts were vortexed for 5 min, then treated with ultrasonication for 10 min. Samples were centrifuged at 12,000 × g at 4 °C for 3 min and the supernatant was removed. The supernatants were collected and filtrated through a membrane with a 0.22-μm pore size (Anpel Laboratory Technologies, Shanghai, China).

The samples were separated on the QTRAP 6500 LC–MS/MS platform (AB Sciex, Framingham, MA, USA) using an ACQUITY BEH C18 column (1.7 µm, 2.1 mm*100 mm) (Waters, Milford, MA, USA). Solvent A was 0.1% formic acid in water and Solvent B was 0.1% formic acid in methanol. The gradient program was as follows: 19:1 Solvent A:B at 0 min; 1:1 Solvent A:B at 6 min; 1:19 Solvent A:B from 12–14 min; and 19:1 Solvent A:B from 14–16 min. The flow rate was 0.35 mL/min, the column temperature was 40 °C, and the injection volume was 2 μL. The electrospray ionization (ESI)-MS/MS conditions were as follows: ion source, ESI + ; source temperature, 550 ℃; ion spray (IS) voltage, 5500 V; curtain gas pressure, 35 psi. Peaks were detected and quantified with MetWare (http://www.metware.cn/).

The integration and correction of chromatographic peaks were carried out by MultiaQuant software (AB SCIEX, Concord, ON, Canada), and metabolite data analysis was conducted by using Analyst 1.6.3 software (AB SCIEX, Concord, ON, Canada). The distinction between groups was maximized by partial least squares-discriminant analysis (OPLS-DA), and based on the OPLS-DA results, the differential metabolites were screened by combining fold change and the variable importance in projection values (VIP). VIP ≥ 1 and fold change ≥ 2 or fold change ≤ 0.5 were set as the selection standard differential metabolites.

### Complementary DNA (cDNA) library construction and RNA sequencing

Total RNA was extracted from the *C. oleifera* petals using the RNAprep Pure Plant Kit (Tiangen, Beijing, China) following the manufacturer’s instructions. The RNA concentration and purity were measured on a NanoDrop 2000 (Thermo Fisher Scientific, Wilmington, DE, USA). RNA integrity was assessed using the RNA Nano 6000 Assay Kit with the Agilent Bioanalyzer 2100 system (Agilent Technologies, Santa Clara, CA, USA). Sequencing libraries were generated using the NEBNext Ultra RNA Library Prep Kit for Illumina (New England Biolabs, Ipswich, MA, USA) following the manufacturer’s instructions. Index codes were added to identify each sample. Clean reads were aligned to the *C. oleifera* reference genome using Hisat2. Successfully aligned sequences were assembled and expression levels calculated with StringTie software to establish a transcriptome library.

### Functional annotation of differential expression genes (DEGs)

DEGs between four petals samples were identified with the ‘DESeq2’ R package (v1.16.1). The thresholds were false discovery rate (FDR)-adjusted *p* < 0.05 and |log2(fold change [FC])|≥ 1. DEG expression patterns were displayed as heat maps, which were generated in R software. Enrichment analyses were conducted in the DEG sets using Gene Ontology (GO, http://www.geneontology.org/) annotation terms and Kyoto Encyclopedia of Genes and Genomes (KEGG, http://www.genome.jp/kegg/) biochemical pathways with the ‘cluster Profiler’ package in R, correcting for gene length bias. GO terms with corrected *p*-values < 0.05 were considered significantly enriched.

### Weighted gene co-expression network analysis

The screened DEGs and differentially accumulated metabolites were applied to build a regulatory network through weighted gene co-expression network analysis (WGCNA) tools on Metware Cloud platform. Furthermore, the correlation coefficients between the hub genes in the modules and the differential metabolites were calculated, then we selected the candidate genes with the correlation values ≥ 0.8 or ≤  − 0.8 with the differential metabolites, and drew an interaction network diagram among the candidate genes and metabolites.

### Cis-acting element analysis

The promoter regions (defined as the 2000-bp regions upstream of the translation start sites) of key genes suspected to be responsive to light or sucrose were analyzed to identify putative cis-acting elements. Each promoter region was analyzed using the tool on the PlantCARE website (https://bioinformatics.psb.ugent.be/webtools/plantcare/html/).

### Quantitative reverse transcription (qRT)-PCR analysis

The primer sequences for qRT-PCR (Table S1) were designed in accordance with the mRNA sequences gained from Integrated DNA Technologies (IDT) website. qRT-PCR was performed on a Step One PULS Real-Time Detection System (ABI, Foster, CA, USA) using the SuperReal fluorescence quantitative premix reagent (SYBR Green) kit (Tiangen, Beijing, China). Gene expression was normalized with the 2-^ΔΔCt^ method using β-actin as the internal control.

## Results

### Flavonoid metabolome profile in *Camellia oleifera* petals

Based on the UPLC-MS/MS detection platform, a total of 372 flavonoid metabolite species were identified, including 26 proanthocyanidins, 8 biflavones, 38 tannins, 22 flavanols, 83 flavonols, 85 flavonoids, 27 anthocyanins, 11 dihydroflavonols, 27 flavanones, 6 aurones, 23 chalcones (including C-glucosylquinochalcones), and 9 others. All details were provided in Table S2. It was observed that samples from different-colored petals were clustered together, indicating that the generated metabolic data were highly reliable. Interestingly, a clear separation was found between white, pink petal samples (W and P) and candy pink, red petals samples (CP and R), suggesting that the metabolites profiles in those four samples were obviously distinct. The results of the heatmap demonstrated that four petal samples with different color were divided into two clusters, the metabolites between W, P, CP and R petals exhibited a different accumulation level (Fig. S1).

**Fig. 2:**
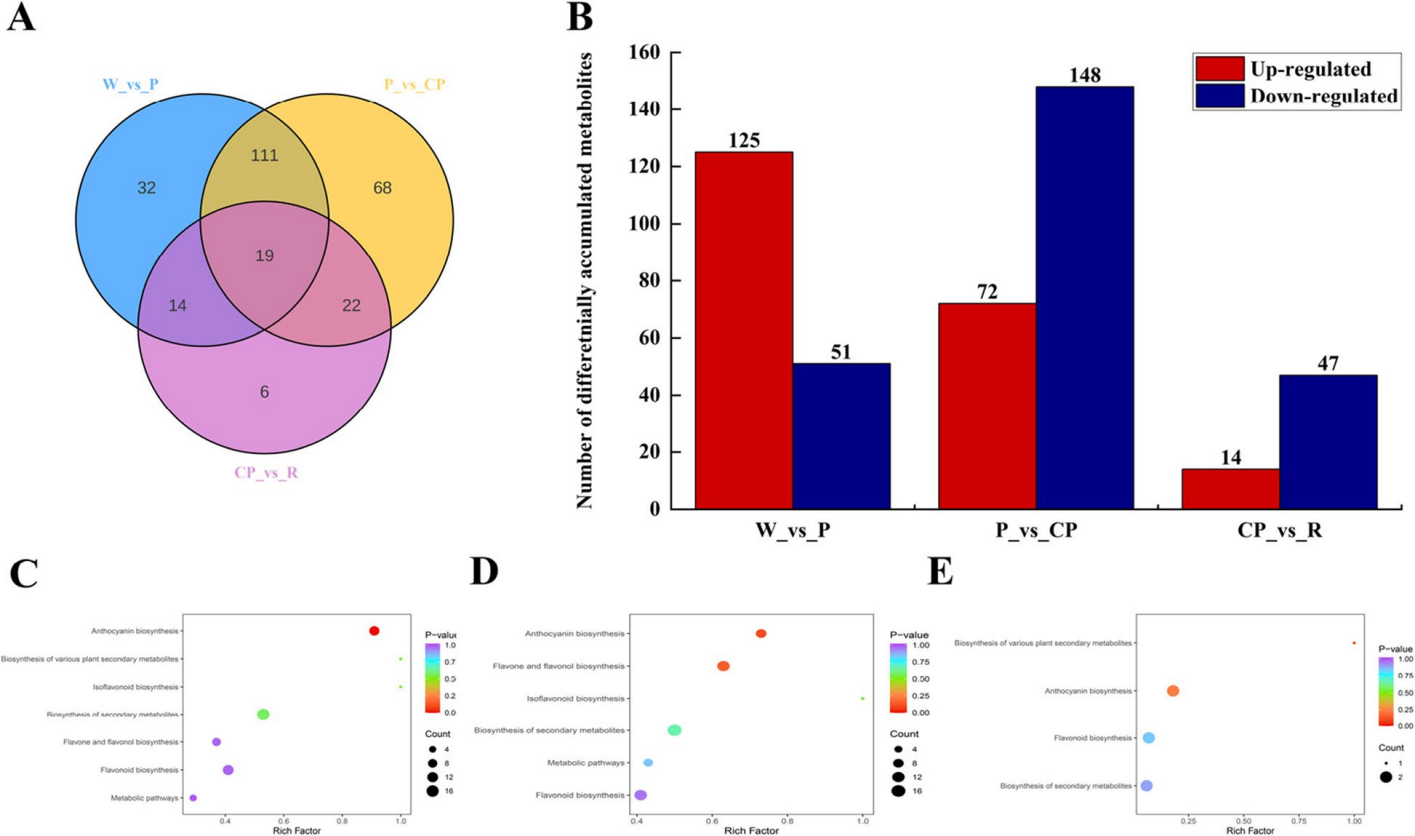
**A** Venn diagram. **B** Numbers of differential metabolites in W, P, CP and R, red indicated up-regulated differential metabolites, and blue indicated down-regulated differential metabolites. **C** W-vs-P; **D** P-vs-CP; **E** CP-vs-R, abscissa indicated the rich factor corresponding to each pathway, ordinate indicated the pathway name, dot color indicated *p*-value, and dot size indicated the number of enriched differential metabolites. We obtained permission to use the KEGG software from the Kanehisa laboratory (Ref: 231,702). W: “*C. yuhsienensis*” petals, P: “*C. reticulate*” petals, CP: “*C. semiserrata*” petals, R: “*C. chekiangoleosa*” petals

The differentially accumulated metabolites (DAMs) between pairwise comparisons among W_vs_P, P_vs_CP, and CP_vs_R were screened by the variable importance in projection values (VIP) ≥ 1 and fold change ≥ 2 or fold change ≤ 0.5. The W_vs_P comparison and P_vs_CP comparison had the largest number of upregulated and down-regulated DAMs (Fig. 2A). Among these comparisons, there were 176 DAMs (125 up-regulated and 51 down-regulated) in the W_vs_P comparison, 220 DAMs (72 up-regulated and 148 down-regulated) in the P_vs_CP comparison, and 61 DAMs (14 up-regulated and 47 down-regulated) in the CP_vs_R comparison, respectively (Fig. 2B). KEDD pathway enrichment analyses were carried out to gain further insights into the biochemical pathway to which the DAMs belonged. The top three enriched KEGG pathway between the 3 comparisons were anthocyanin biosynthesis, flavonoid biosynthesis, and flavone and flavonol biosynthesis (Fig. 2C-E). Given the role of anthocyanins and flavonoids in petals coloration, we deduced that the DAMs in anthocyanin biosynthesis pathway and flavonoid biosynthesis pathway might be likely the key metabolites underlying the variations in *C. oleifera* petals.

### Analysis of related metabolites in anthocyanin synthesis pathway

A total of 27 anthocyanins were identified in *C. oleifera* petals, including cyanidin, peonidin, pelargonidin, delphinidin and malvidin (Table 1). There were 22 differentially accumulated anthocyanins (22 up-regulated) in W_vs_P comparison (Table S3), 22 differentially accumulated anthocyanins (8 up-regulated and 14 down-regulated) in P_vs _CP comparison (Table S4), and 2 differentially accumulated anthocyanins (1 up-regulated and 1 down-regulated) in CP_vs_R comparison (Table S5). Among them, cyanidin-3-O-(6''-O-p-Coumaroyl) glucoside, cyanidin-3-O-arabinoside, pelargonidin-3-O-glucoside, delphinidin-3-O-galactoside and peonidin-3-O-glucoside in P petals were increased by 458.44-, 263.94-, 191.00-, 166.80-, 159.34-fold, respectively, compared with those in W petals. Cyanidin-3-O-glucoside, cyanidin-3-O-galactoside, cyanidin-3-O-rutinoside, and cyanidin-3-O-(6''-O-malonyl) glucoside in CP petals were increased by 11.92-, 5.03-, 3.80-, and 3.72-fold, respectively, compared with that in P petals, while cyanidin-3-O-(6''-O-p-Coumaroyl) glucoside were decreased by 0.28-fold. Peonidin-3-O-glucoside in R petals was 4.63-fold higher than that in CP petals, and cyanidin-3-O-(6''-O-malonyl) glucoside was decreased by 0.20-fold. Cyanidin-3-O-(6''-O-p-Coumaroyl) glucoside was the main color constituent in pink petals, cyanidin-3-O-glucoside, cyanidin-3-O-galactoside, cyanidin-3-O-rutinoside, and cyanidin-3-O-(6''-O-malonyl) glucoside were the main anthocyanins in candy pink petals, and peonidin-3-O-glucoside was the important coloring substance in the red petals. Cyanidin-3-O-glucoside, cyanidin-3-O-galactoside, cyanidin-3-O-rutinoside, peonidin-3-O-glucoside, cyanidin-3-O-(6''-O-malonyl) glucoside, and cyanidin-3-O-(6''-O-p-Coumaroyl) glucoside were speculated to be important anthocyanins for the coloration of *C. oleifera* petals, and the difference in content might be the main reasons for the difference in *C. oleifera* petals with different color.

**Table 1:** Differentially accumulated anthocyanins in four *C. oleifera* petals

Compounds	Molecular weight (Da)	Ion abundance			
		W	P	CP	R
Cyanidin-3-O-(6''-O-malonyl) glucoside	5.35E + 02	6.11E + 04	4.99E + 05	1.86E + 06	3.63E + 05
Cyanidin-3,5-O-diglucoside	6.11E + 02	2.23E + 03	5.87E + 06	7.78E + 05	7.43E + 05
Cyanidin-3-O-gentiobioside	6.11E + 02	1.12E + 03	5.70E + 06	1.08E + 05	1.66E + 05
Cyanidin-3-O-sambubioside-5-O-glucoside	7.43E + 02	2.55E + 03	3.00E + 05	1.51E + 04	1.06E + 04
Cyanidin-3-O-xyloside	4.19E + 02	9.00E + 00	1.70E + 05	7.65E + 05	1.38E + 06
Pelargonidin-3-O-rutinoside	5.79E + 02	7.75E + 06	6.41E + 06	9.24E + 06	1.27E + 07
Cyanidin-3-O-glucoside	4.49E + 02	2.78E + 03	1.51E + 06	1.80E + 07	2.35E + 07
Malvidin-3-O-(6''-O-malonyl) glucoside	5.79E + 02	3.59E + 05	2.99E + 05	3.78E + 05	3.20E + 05
Cyanidin-3-O-galactoside	4.49E + 02	3.76E + 04	1.19E + 07	6.00E + 07	7.15E + 07
Cyanidin-3-O-(6''-O-caffeoyl-2''-O-xylosyl) glucoside	7.43E + 02	3.23E + 03	6.66E + 06	9.00E + 00	5.15E + 03
Cyanidin-3-O-(2''-O-xylosyl) glucoside-5-O-glucoside	7.43E + 02	3.86E + 03	3.33E + 05	1.47E + 04	1.07E + 04
Pelargonidin-3,5-O-diglucoside	5.95E + 02	4.57E + 04	2.46E + 06	6.62E + 04	9.14E + 04
Cyanidin-3-O-(6''-O-p-Coumaroyl) glucoside	5.95E + 02	3.92E + 04	1.80E + 07	4.95E + 06	6.52E + 06
Cyanidin-3-O-(6''-O-caffeoyl) glucoside	6.11E + 02	9.45E + 03	2.42E + 06	1.30E + 05	6.50E + 04
Peonidin-3-O-glucoside	4.63E + 02	9.89E + 03	1.58E + 06	2.75E + 06	1.28E + 07
Cyanidin-3-O-arabinoside	4.19E + 02	9.58E + 02	2.53E + 05	9.04E + 05	1.70E + 06
Pelargonidin-3-O-glucoside	4.33E + 02	5.79E + 04	1.11E + 07	6.60E + 06	6.27E + 06
Peonidin-3-O-(6''-O-p-coumaroyl) glucoside	6.09E + 02	2.79E + 04	3.46E + 06	6.16E + 05	6.42E + 05
Delphinidin-3-O-sambubioside	5.97E + 02	2.35E + 04	3.11E + 05	1.63E + 04	2.14E + 04
Peonidin-3-O-rutinoside	6.09E + 02	2.94E + 03	4.20E + 04	2.91E + 05	3.48E + 05
Delphinidin-3-O-galactoside	4.65E + 02	2.44E + 04	4.07E + 06	8.78E + 04	1.25E + 05
Cyanidin-3-O-(6''-O-acetyl) glucoside-5-O-glucoside	6.53E + 02	8.54E + 02	3.16E + 05	5.69E + 03	9.00E + 00
Pelargonidin-3-O-glucoside-5-O-arabinoside	5.65E + 02	4.94E + 03	9.77E + 05	3.03E + 03	2.11E + 03
Cyanidin-3-O-(6''-O-acetyl) glucoside	4.91E + 02	3.40E + 03	3.61E + 05	6.19E + 05	6.61E + 05
Cyanidin-3-O-sophorotrioside	7.73E + 02	9.00E + 00	3.47E + 05	1.81E + 04	1.88E + 04
Delphinidin-3-O-glucoside (Mirtillin)	4.65E + 02	2.50E + 04	1.80E + 06	6.32E + 06	5.03E + 06
Cyanidin-3-O-rutinoside (Keracyanin)	5.95E + 02	4.92E + 04	3.37E + 06	1.28E + 07	9.57E + 06

W: “*C. yuhsienensis*” petals, P: “*C. reticulate*” petals, CP: “*C. semiserrata*” petals, R: “*C. chekiangoleosa*” petals

### Transcriptome sequencing and identification of DEGs in *C. oleifera* petals

To further elucidate the molecules mechanisms of *C. oleifera* petals coloration, Deiiovo-RNAseq was performed to investigated the variations in genes expression profiles among the four petals samples. The transcriptome profiling was performed on twelve libraries (4 petals × 3 biological repeats) yield a total of 95.20 Gb clean data with 92.00% or more of bases scoring Q30 (Table 2), with mapping rates between 75.45% and 81.07%. In all, 60,720 genes comprising 27,429 known genes and 33,291 novel genes were detected from the 12 libraries (Table S6). PCA results of the 12 samples were clustered together, indicating the transcriptome sequencing data was reliable. Similar to the metabolites, there was an obvious separation between white-, pink-colored petal samples and candy pink-, red-colored petals samples, implying the changes in metabolites in different color petals were tightly governed by differential expression genes.

**Table 2:** Sequencing and quality statistics

Sample	Clean Reads	Clean Base (Gb)	Error Rate (%)	Q20 (%)	Q30 (%)	GC Content (%)
W-1	49,668,862	7.45	0.03	97.58	93.14	44.58
W-2	48,129,746	7.22	0.03	97.40	92.80	44.83
W-3	49,468,544	7.42	0.03	97.03	92.17	45.02
P-1	55,277,188	8.29	0.03	97.41	92.72	44.94
P-2	51,166,048	7.67	0.03	97.20	92.24	44.98
P-3	59,686,126	8.95	0.03	97.38	92.63	44.94
CP-1	52,244,350	7.84	0.03	97.44	92.79	44.55
CP-2	53,492,038	8.02	0.03	97.49	92.91	44.58
CP-3	47,874,262	7.18	0.03	97.60	93.15	44.64
R-1	54,281,030	8.14	0.03	97.46	92.9	44.86
R-2	48,963,938	7.34	0.03	97.50	92.86	44.65
R-3	64,527,812	9.68	0.03	97.61	93.15	44.96

W: “*C. yuhsienensis*” petals, P: “*C. reticulate*” petals, CP: “*C. semiserrata*” petals, R: “*C. chekiangoleosa*” petals

**Fig. 3:**
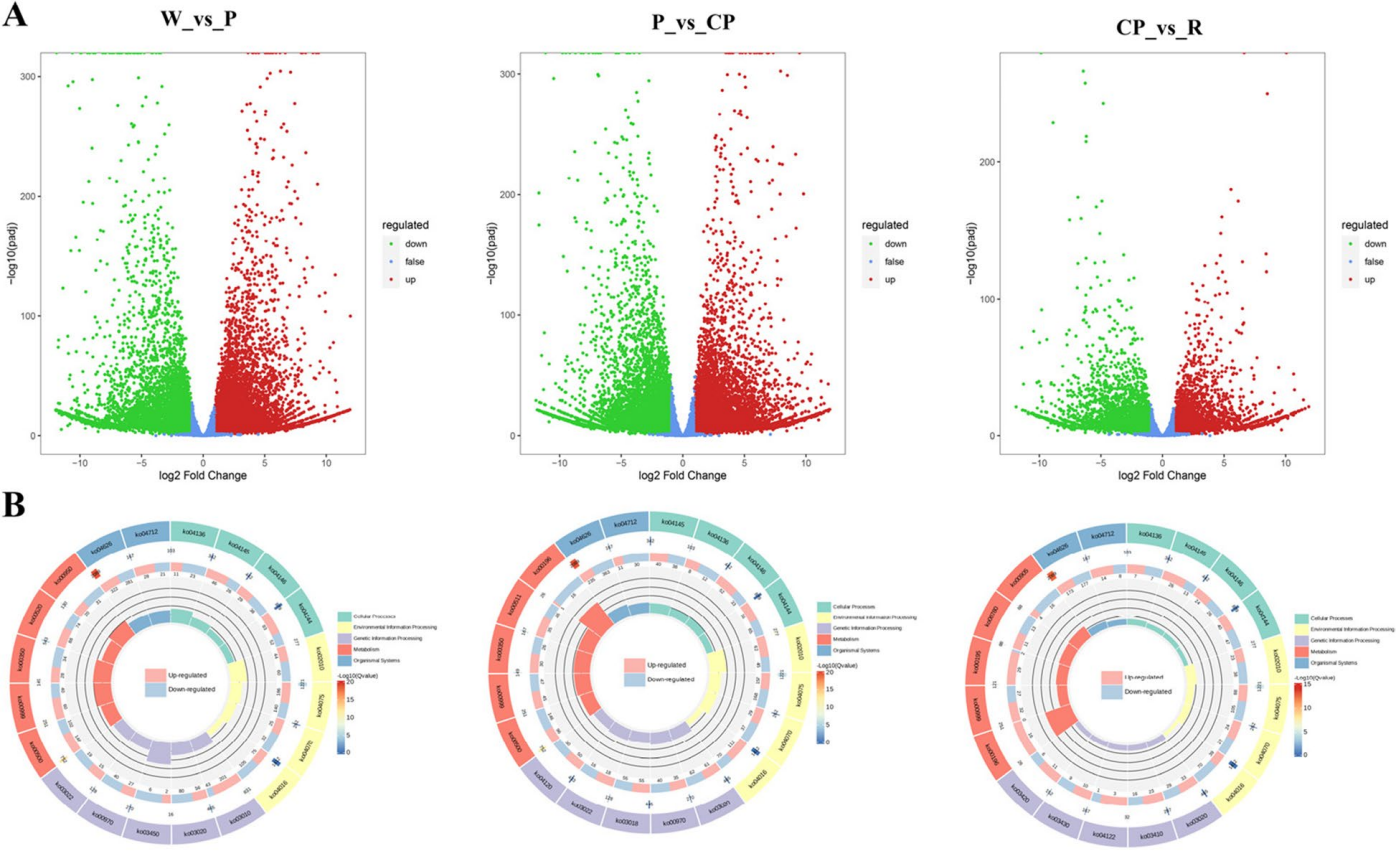
Differential expressed genes (DEGs) in four *C. oleifera* petals. **A** Volcano plots displaying the up-regulated (red color), down-regulated (green color) and no-regulated genes (blue color) in the comparsion of W_vs_P, P_vs_CP, and CP_vs_R, **B** Top KEGG terms contributed by the DEGs in the comparsion of W_vs_P, P_vs_CP, and CP_vs_R. W: “*C. yuhsienensis*” petals, P: “*C. reticulate*” petals, CP: “*C. semiserrata*” petals, R: “*C. chekiangoleosa*” petals

Using an FDR-adjusted *p*-value threshold of 0.05 and a |log2(fold change)| threshold of 1, selected pairwise comparisons among the W, P, CP, and R petals samples yielded a total of 24,334 DEGs. There were 15,375 DEGs (8318 up-regulated and 7057 down-regulated) in W_vs_P comparison, 15,157 DEGs (7532 up-regulated and 7625 down-regulated) in P_vs_CP comparison, and 8037 DEGs (3879 up-regulated and 4158 down-regulated) in CP_vs_R comparison (Fig. 3A). There were 6099, 4374, and 1963 unique DEGs in the comparison of W_vs_P, P_vs_CP, and CP_vs_R, respectively. The results demonstrated that 2337 genes were differentially expressed in the common across the three comparisons, suggesting that those DEGs might be the key genes related to color expression of *C. oleifera* petals.

To understand the functions of DEGs in each of the three comparison groups, we conducted GO annotation enrichment analysis. Differential GO clustering analysis focused on three major categories: cellular component, biological process, and molecular function. Enriched molecular function terms included “UDP-glucosyltransferase activity”, “glucosyltransferase activity”, and enriched biological process terms included “phenylpropanoid metabolic process”, and “secondary metabolite biosynthetic process”. A total of 168 and 159 unigenes were involved in the phenylpropanoid metabolic process and secondary metabolite biosynthetic process in the biological process, and 155 unigenes had UDP-glucosyltransferase activity in the molecular function.

KEGG pathway enrichment analyses were applied to gain further into the biochemical pathway to which the DEGs belonged. In the comparisons of W_vs_P, enriched pathways included “plant hormone signal transduction”, “ABC transporters”, “starch and sucrose metabolism”, “phenylpropanoid biosynthesis”, and “biosynthesis of secondary metabolites”. The terms of “plant hormone signal transduction”, “amino sugar and nucleotide sugar metabolism”, “biosynthesis of various plant secondary metabolites”, “phenylalanine metabolism”, and “ABC transporters” were significantly enriched in the comparisons of P_vs_CP and CP_vs_R (Fig. 3B). We focused on the several pathways associated with anthocyanins synthesis for further analyses, because they were key components of environmental adaptation and specialized metabolite biosynthesis: “plant hormone signal transduction”, “flavonoid biosynthesis”, “phenylpropanoid biosynthesis”, and “anthocyanins biosynthesis”. Based on the selected DEGs enriched by KEGG pathway combined with enriched GO functional annotations, we screeded the DEGs related to anthocyanins biosynthesis, including the *PAL*, *CHS*, *CHI*, *F3H*, *F3′H*, *F3′5′H*, *DFR*, *ANS*, and *UFGT*, which were annotated as members of the flavonoid biosynthesis and anthocyanin biosynthesis pathways.

### The key DEGs related to anthocyanin biosynthesis pathway

**Fig. 4:**
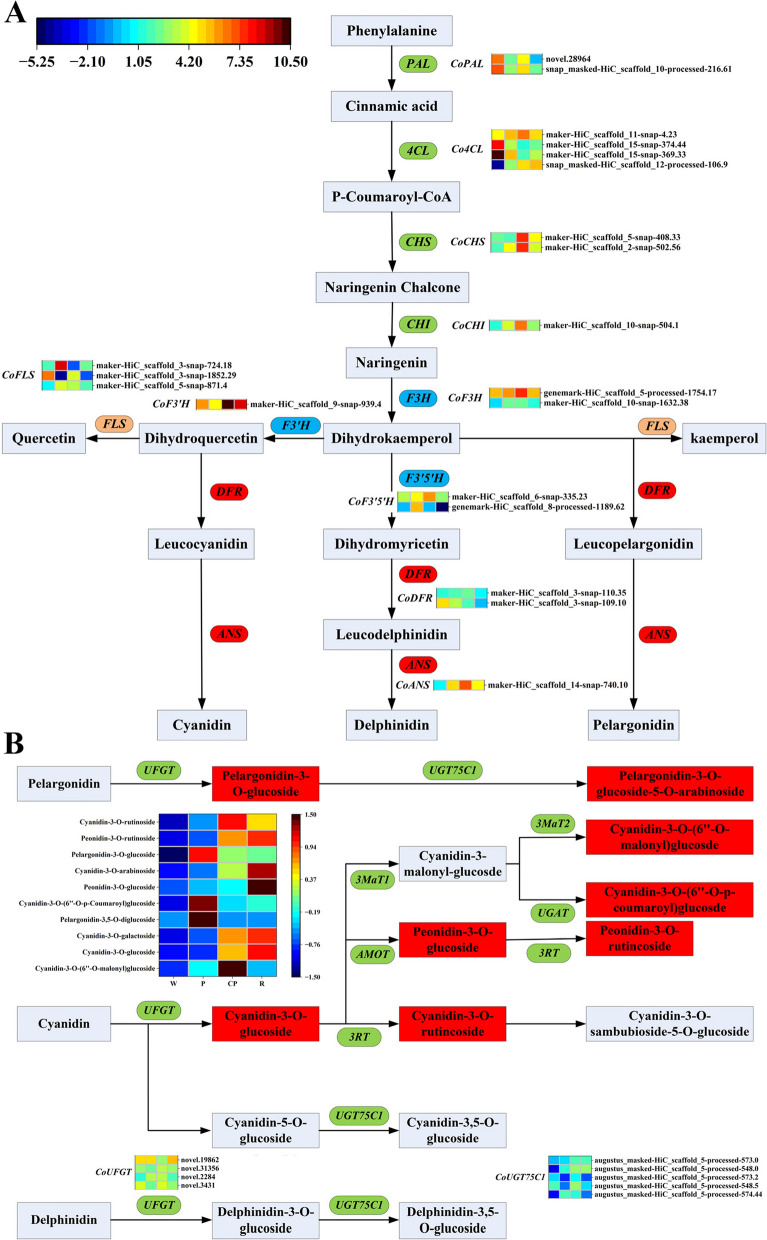
Expression levels of structural genes involved in anthocyanin biosynthesis pathway in *C. oleifera* petals. **A** The identification of all candidate structural genes involved in phenylpropanoid biosynthesis, flavonoid biosynthesis. The color from blue to red in the heatmap indicated the expression levels of structural genes ranging from low to high. **B** The identification of all candidate structural genes involved in anthocyanins biosynthesis. Note: W: “*C. yuhsienensis*” petals, P: “*C. reticulate*” petals, CP: “*C. semiserrata*” petals, R: “*C. chekiangoleosa*” petals. *CoPAL*, phenylalanine ammonia lyase; *Co4CL*, 4-coumarate: CoA ligase; *CoCHS*, chalcone synthase; *CoCHI*, chalcone isomerase; *CoF3H*, flavanone 3-hydroxylase; *CoF3′H*, flavonoid 3′-hydroxylase; *CoF3′5′H*, flavonoid 3′,5′-hydroxylase; *CoDFR*, dihydroflavonol 4-reductase; *CoANS*, anthocyanidin synthase; *CoUFGT*, anthocyanidin 3-O-glucosyltransferase; *CoUGT75C1*, anthocyanidin 3-O-glucoside 5-O-glucosyltransferase

A total of 32 structural DEGs related to phenylpropanoid biosynthesis, flavonoid biosynthesis, and anthocyanin biosynthesis pathways were screened, including *CoPAL*, *Co4CL*, *CoCHS*, *CoCHI*, *CoF3H*, *CoFLS*, *CoF3'H*, *CoF3′5'H*, *CoDFR*, *CoLAR*, *CoANS*, *CoUFGT*, and *CoUGT75C1*, and the expression levels of each gene could be viewed in Table S7. The structural genes *CoPAL*, *Co4CL*, *CoCHS*, and *CoCHI* were involved in the initial stage of anthocyanin biosynthesis process. It was clearly found that two *CoPAL* genes (snap_masked-HiC_scaffold_10-processed-216.61 and novel.28964) exhibited a high expression in W petals and a low expression in P, CP, and R petals, while those two *CoPAL* genes were upregulated from P teals to CP petals. Two *CoCHS* genes (maker-HiC_scaffold_2-snap-502.56 and maker-HiC_scaffold_5-snap-408.33), and one *CoCHI* gene (maker-HiC_scaffold_10-snap-504.1) were downregulated from CP petals to R petals, and one *CoCHS* genes (maker-HiC_scaffold_2-snap-502.56) and the *CoCHI* gene were upregulated in P and CP petals. Dihydrokaempferol was generated under the catalysis of the *CoF3H* gene. Two *CoF3H* genes were highly expressed in CP petals (maker-HiC_scaffold_10-snap-1632.38 and genemark-HiC_scaffold_5-processed-1754.17), one that was differentially expressed only in P petals (maker-HiC_scaffold_10-snap-1632.38), one that was differentially expressed only in CP petals (genemark-HiC_scaffold_5-processed-1754.17), and two that were differentially expressed only in R petals. The *CoF3′H* and *CoF3′5′H* genes were engaged in the key branch pathways in anthocyanin synthesis pathway, which determined what type of anthocyanin was produced. One *CoF3′H* gene (maker-HiC_scaffold_9-snap-939.4) was downregulated frow W petals to P petals, and was upregulated from P to CP, and was downregulated from CP petals to R petals. One *CoF3′5′H* genes (genemark-HiC_scaffold_8-processed-1189.62) was presented with high expression levels in P petals, and One *CoF3′5′H* genes (maker-HiC_scaffold_6-snap-335.23) was upregulated from CP petals to P petals, and was downregulated from CP petals to R petals (Fig. 4A).

*CoDFR* is mainly responsible for catalyzing the conversion of dihydroflavonol to leucocyanidin, leucodelphinidin, and leucopelargonidin. Here, two *CoDFR* genes were identified (maker-HiC_scaffold_3-snap-109.10 and maker-HiC_scaffold_3-snap-110.35), in which one *CoDFR* gene (maker-HiC_scaffold_3-snap-109.10) was highly expressed in W petals and was downregulated in P, CP and R petals, and one *CoDFR* gene (maker-HiC_scaffold_3-snap-110.35) was upregulated in P and CP petals. And, one *CoANS* gene (maker-HiC_scaffold_14-snap-740.10) was upregulated in P, CP, and R petals compared to W petals, while was downregulated from CP petals to R petals. *CoUFGT* and *CoUGT75C1* are the last enzyme encoded by structural genes, which could catalyze unstable anthocyanins into anthocyanins. Four *CoUFGT* genes were (novel.3431, novel.2284, novel.31356, and novel.19862) identified, and one gene (novel.2284) was upregulated from W petals to P petals, two genes (novel.3431 and novel.31356) were upregulated from P petals to CP petals. One *UFGT* gene (novel.19862) was upregulated from CP petals to R petals, and two genes (novel.3431 and novel.2284) were downregulated. Three *CoUGT75C1* genes (augustus_masked-HiC_scaffold_5-processed-574.44, augustus_masked-HiC_scaffold_5-processed-548.0, and augustus_masked-HiC_scaffold_5-processed-573.0) were upregulated from W petals to P petals, and two genes (augustus_masked-HiC_scaffold_5-processed-548.5 and augustus_masked-HiC_scaffold_5-processed-573.2) were downregulated. Four *CoUGT75C1* genes (augustus_masked-HiC_scaffold_5-processed-548.5, augustus_masked-HiC_scaffold_5-processed-573.2, augustus_masked- HiC_scaffold_5-processed-548.0, and augustus_masked-HiC_scaffold_5-processed-573.0) were upregulated in CP petals. Three *CoUGT75C1* genes (augustus_masked-HiC_scaffold_5-processed-574.44, augustus_masked-HiC_scaffold_5-processed-548.5, and augustus_masked-HiC_scaffold_5-processed-573.2) were downregulated from CP petals to R petals (Fig. 4B).

### Identification of transcription factors (TFs) involved in anthocyanin biosynthesis by WGCNA

**Fig. 5:**
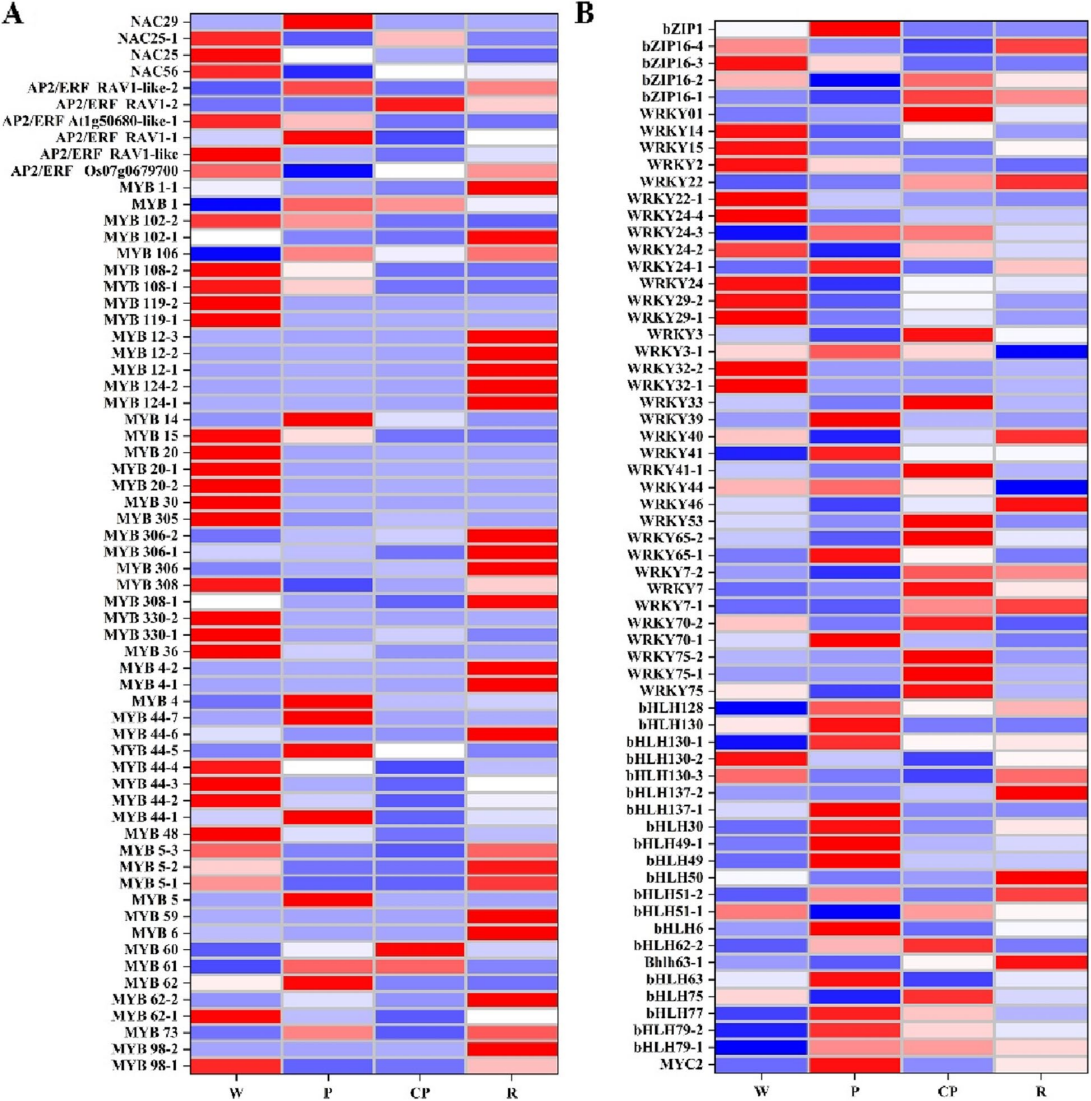
Analysis of TFs linked to anthocyanins biosynthesis. **A** The heatmap of the expression of MYB, AP2/ERF, and NAC, and (**B**) The heatmap of expression of bHLH, WRKY, and bZIP. W: “*C. yuhsienensis*” petals, P: “*C. reticulate*” petals, CP: “*C. semiserrata*” petals, R: “*C. chekiangoleosa*” petals

Transcription factors (TFs) are the important regulators of anthocyanins biosynthesis and accumulation via controlling the structural genes expression. In our study, a total of 1025 TFs were identified in four *C. oleifera* petals based on the transcriptome annotation results. The TFs classified results displayed that the TFs belonged to MYB, bHLH, WRKY, AP2/ERF, bZIP, and NAC family, and the *MYB*, *bHLH*, and *WRKY* were the top three TFs involved in anthocyanins biosynthesis, The differential expression levels of MYB, AP2/ERF, and NAC TFs were described in Fig. 5A, and the differential expression levels of bHLH, WRKY, and bZIP TFs were described in Fig. 5B. These TFs might directly regulate the anthocyanins biosynthesis or indirectly affect the anthocyanins biosynthesis by regulating structural genes.

**Fig. 6:**
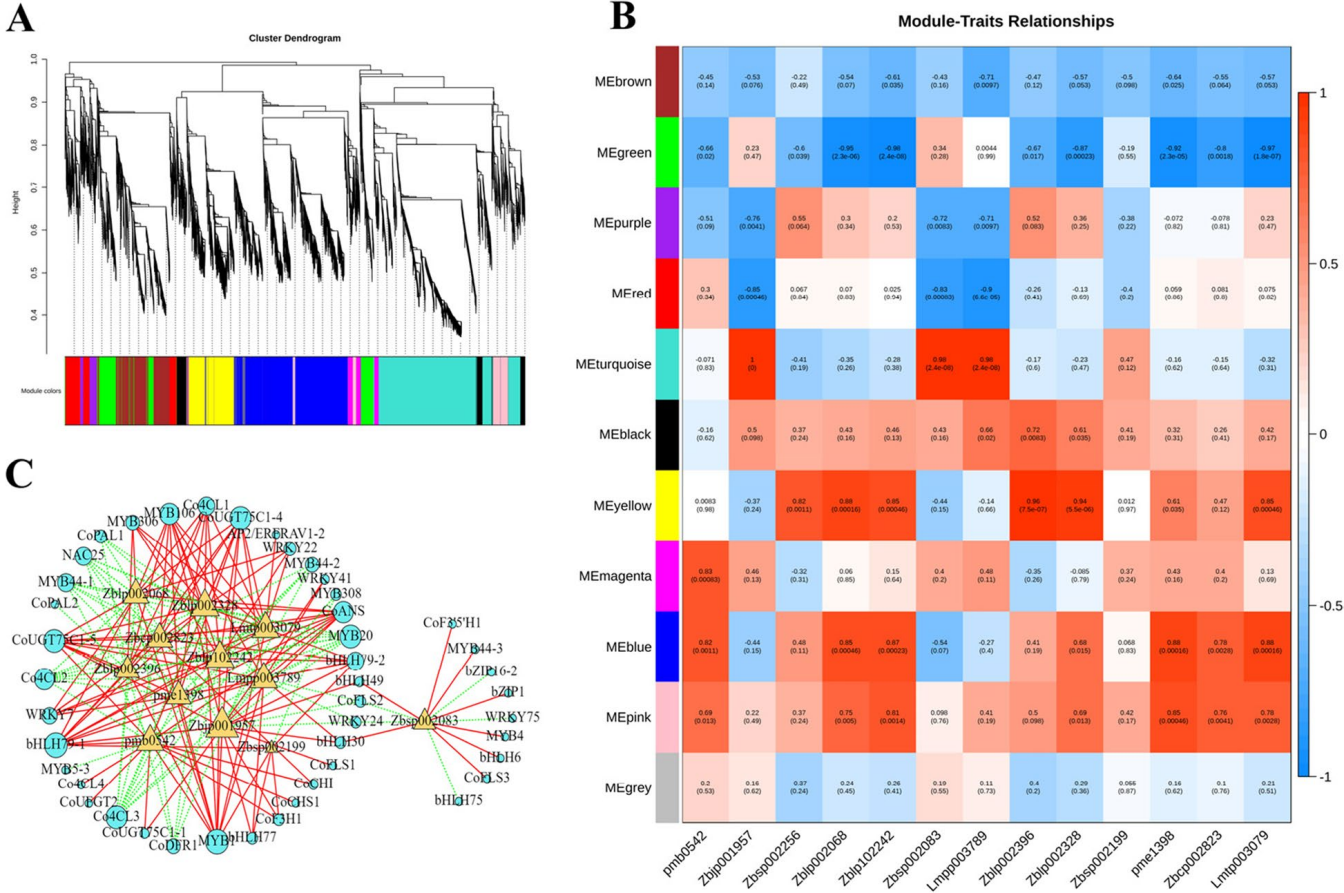
**A** The cluster dendrogram results of 11 genes expression modules, present with different colors. **B** The relationship analysis between genes module and 13 anthocyanins. **C** The correlation network diagram between candidates and anthocyanin. Red solid lines represented the promotion of synthesis, while green dotted lines represented inhibition of accumulation. Red circles represented anthocyanins and blue triangles represented hub genes. pmb0542, Cyanidin-3-O-(6''-O-malonyl) glucoside; Zbjp001957, Cyanidin-3,5-O-diglucoside; Zbsp002256, Pelargonidin-3-O-rutinoside; Zblp002068, Cyanidin-3-O-glucoside; Zblp102242, Cyanidin-3-O-galactoside; Zbsp002083, Pelargonidin-3,5-O-diglucoside; Lmpp003789, Cyanidin-3-O-(6''-O-p-Coumaroyl) glucoside; Zblp002396, Peonidin-3-O-glucoside; Zblp002328, Cyanidin-3-O-arabinoside; Zbsp002199, Pelargonidin-3-O-glucoside; pme1398, Delphinidin-3-O-glucoside; Zbcp002823, Cyanidin-3-O-rutinoside; Lmtp003079, Peonidin-3-O-rutinoside

We next sought to more comprehensively identify the specific transcription factors that regulated anthocyanin structural genes in *C. oleifera* petals. WGCNA was applied to conduct using the FPKM values of 24,334 DEGs and 13 anthocyanins as source data. 11 modules were identified in the cluster dendrogram, named as brown, green, purple, red, turquoise, black, yellow, magenta, blue, pink, and grey modules (Fig. 6A). The relationships result between the gene modules and anthocyanins revealed that the blue, pink, yellow, red, and turquoise pink modules had high correlation with anthocyanins (Fig. 6B). To describe the relationship between genes in these five modules and 13 anthocyanins, a heat map was performed using the correlation coefficient values of genes and anthocyanins in these modules. In the pink module, the co-expressed genes were highly expressed in P, CP and R petals (Fig. S2A). The heatmap of brown module (Fig. S2B), the turquoise module (Fig. S2C) and the yellow module (Fig. S2D) suggested that the co-expressed genes were only highly expressed in W petals, in P petals and in R petals, respectively.

Moreover, a directed interaction network was built based on the correlation coefficient between the candidate genes in the blue, pink, yellow, red, and turquoise pink modules and 13 anthocyanins content, with the |correlation coefficient|> 0.8 and *p* < 0.05. These five modules contained a total of 14 MYBs, 9 bHLHs, 10 WRKYs, 2 AP2/ERFs, 3 bZIPs, and 1 NAC. As described in Fig. 6C, 25 TFs (|correlation coefficient|> 0.8, *p* < 0.05) were screened out to establish a regulatory network with 12 anthocyanins. The expression levels of *CoMYB1*, *CoMYB4*, *CoMYB44-3*, *CobHLH30*, *CobHLH 77*, *CobHLH 79–1*, *CoWRKY7*, and *CoWRKY22* exhibited a significant positive correlation with one or more anthocyanins (*p* < 0.05), while *CoMYB20*, *CoMYB44-1*, *CoMYB44-2*, *CobHLH75*, *CoNAC25*, and *CobZIP16-2* displayed a significant negative correlation with one or more anthocyanins. The network diagram also revealed that 12 anthocyanins were positively regulated by the structural genes (*CoPAL*, *Co4CL*, *CoCHS*, *CoCHI*, *CoF3H*, *CoFLS*, *CoDFR*, *CoANS*, *CoUFGT*, *CoUGT75C1*). Among them, five structural genes (*Co4CL1*, *CoF3H1*, *CoANS*, *CoUGT75C1-4*, and *CoUGT75C1-5*) were significantly positively correlated with the associated anthocyanin metabolites.

Based on the functional domains present in MYBs and bHLHs and their suspected roles inanthocyanin biosynthesis, we next examined anthocyanin structural genes for evidence of regulation by MYBs or bHLHs. Indeed, the promoter analysis of these candidate genes revealed that *CoF3'H* (Table S8) and *CoANS* (Table S9) contained MYB and bHLH binding sites. We therefore hypothesized that the candidate transcription factors bHLHs (*CobHLH30*, *CobHLH77*, and *CobHLH79*) and MYBs (*CoMYB1*, *CoMYB4*, *CoMYB20*, and *CoMYB44-3*) might specifically bind to *CoF3'H* and *CoANS* through these binding sites, thereby accurately regulated the structural genes in the transcriptional regulation of anthocyanin biosynthesis in *C. oleifera* petals. The above results indicated that the significant difference in the content of 12 anthocyanins might be caused by the above five structural genes in *C. oleifera* petals, and those five structural genes might be regulated by those MYBs and bHLHs transcription factors.

### Verification of the Results in RNA-seq by qRT-PCR

**Fig. 7:**
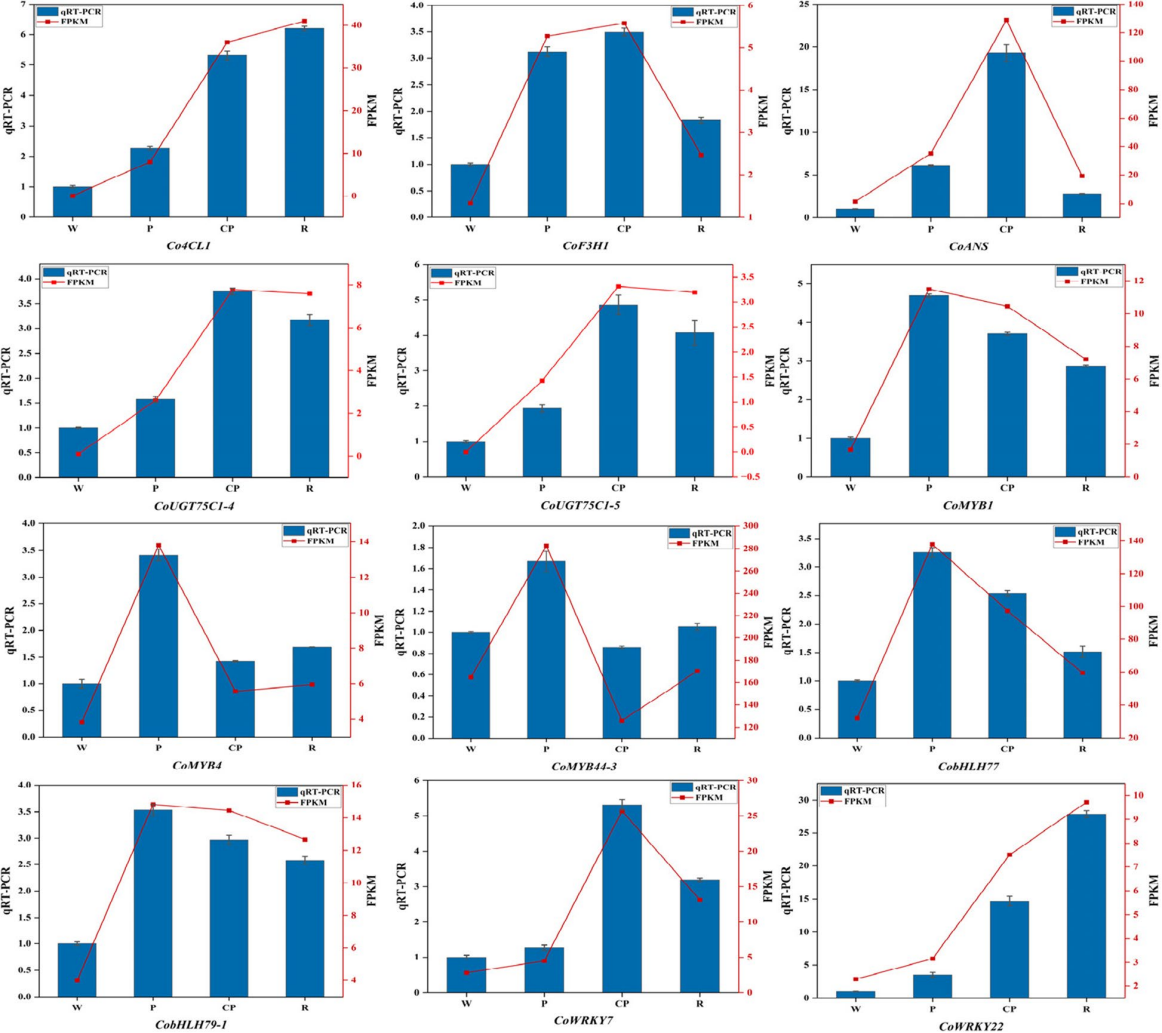
Validation of selected candidate gene expression levels with quantitative reverse transcription PCR. W: “*C. yuhsienensis*” petals, P: “*C. reticulate*” petals, CP: “*C. semiserrata*” petals, R: “*C. chekiangoleosa*” petals

To validate the RNA-seq results, several genes were quantified with qRT-PCR: five structural genes linked to anthocyanin biosynthesis (*Co4CL1*, *CoF3H1*, *CoANS*, *CoUGT75C1-4*, and *CoUGT75C1-5*), three MYBs (*CoMYB1*, *CoMYB4*, and *CoMYB44-3*), three bHLHs (*CobHLH77* and *CobHLH79-1*), and two WRKYs (*CoWRKY7* and *CoWRKY22*). The relative expression levels of these 12 genes were normalized based on the β-actin expression (Fig. 7). Further analysis revealed that qRT-PCR results were fully consistent with and validated the reliability of the RNA-seq data. Therefore, it could be speculated that the above 12 candidate genes might be involved in the formation of flower color diversity in *C. oleifera* petals.

## Discussion

### Anthocyanin identification in the four *C. oleifera* petals

The presence of anthocyanins in petals and fruits is a fundamental factor in the formation of flower color, and its contents and types in petals will definitely change the color of flowers [26, 27]. As secondary metabolites, anthocyanins might directly affect the color of flowers and fruits [27, 28]. For example, red jujube peels were rich in malvidin 3-O-glucoside and delphinidin 3-O-glucoside [12]. Cyanidin-3-O-(6″-Omalonyl) glucoside, cyanidin-3-O-(6″-O-p-coumaroyl-2″-Oxylosyl) glucoside, cyanidin-3-O-arabinoside, cyanidin-3-Ogalactoside, and cyanidin-3-O-glucoside were contributed to the red and pink petal flower of *Camellia reticulata* [6]. Considering that anthocyanins determine the color of flowers, flowers with bright colors have excellent ornamental value, how to breed flowers with specific colors has become a new research issue. Similar to others flowers, the petals of *C. oleifera* have long flowering period and bright color and can be used as ornamental flowers [29]. Thus, the *C. oleifera* petals might be selected as new resource in ornamental flowers. However, no researches or reports had focused on the anthocyanins in *C. oleifera* petals. To reveal the differences of anthocyanins in *C. oleifera* petals with different flower color, the metabolomic analysis of anthocyanins by UPLC‒MS/MS was conducted.

In our study, the UPLC‒MS/MS results displayed that the main anthocyanins in *C. oleifera* petals were pelargonidin-, cyanidin-, delphinidin-, and peonidin- derivatives, but the malvidin- and petunidin- derivatives were not detected, indicating that pelargonidin-, cyanidin-, delphinidin-, and peonidin- derivatives were related to the coloration of *C. oleifera* petals. Our findings were consistent with the results of Fu et al. (2021), who found that pelargonidin, cyanidin, delphinidin, and peonidin were the main anthocyanins components in *C. japonica* petals. And, 27 anthocyanins components were identified in the four *C. oleifera* petals with significant variation in petals color. 13 anthocyanins differentially accumulated among four *C. oleifera* petals based on the anthocyanins content in each sample, indicating that those 13 anthocyanins were contributed to the different petal colors in *C. oleifera*. Among those anthocyanins, peonidin-3-O-glucoside with highest content was responsible for the coloration of red petals. The anthocyanins accumulated in candy pink petals were cyanidin-3-O-glucoside, cyanidin-3-O-galactoside, and cyanidin-3-O-rutinoside, and cyanidin-3-O-(6''-O-malonyl) glucoside, which might lead to candy pink phenotypes. Cyanidin-3-O-(6''-O-p-Coumaroyl) glucoside was the main color substance for the pink petals of *C. oleifera*. Our findings supported the results of *C. japonica* petals, which proposed that cyanidin-3-O-(6''-O-malonyl) glucoside was the primary anthocyanins in the pink flowers. Reports on the *Camellia* document that cyanidin-3-O-glucoside, cyanidin-3-O-galactoside, cyanidin-3-O-rutinoside, peonidin-3-O-glucoside, cyanidin-3-O-(6''-O-malonyl) glucoside, and cyanidin-3-O-(6''-O-p-Coumaroyl) glucoside accumulated in pink and red petals but not in white flower, which indicated that these compounds played important role in the coloration. The accumulation of cyanidin-3-O-glucoside, cyanidin-3-O-galactoside, cyanidin-3-O-rutinoside, peonidin-3-O-glucoside, cyanidin-3-O-(6''-O-malonyl) glucoside, and cyanidin-3-O-(6''-O-p-Coumaroyl) glucoside could be the main coloring substances for the color difference in four *C. oleifera* petals. All these results indicated that the type and content of anthocyanin compounds, particularly cyanidin-based and peonidin-based anthocyanins, were the main factors for the formation of white, pink, candy pink and red petals of *C. oleifera*.

### Key structural genes responsible for anthocyanin biosynthesis in *C. oleifera* petals

Previous researches have approved that the structural genes such as *PAL*, *C4H*, *4CL*, *CHS* and *CHI* were involved in the early enzymatic reaction in the process of anthocyanin biosynthesis, and *F3′H*, *F3′5′H*, *DFR*, *ANS*, *UFGT* and *UGT75C1* are the downstream genes of anthocyanin synthesis [30, 31]. 37 candidate structural genes (especially *CjANS* and *Cj4CL*) regulating anthocyanin accumulation were identified in *C. japonica* petals [6]. The structural genes *F3H*, *F3′H*, *UFGT*, and *GST* involved in the anthocyanins biosynthesis pathway were significantly upregulated in red pericarp of *Dimocarpus longan* [32]. Three *UFGT* genes linked to the anthocyanin accumulation were significantly increased and could contribute to the jujube fruit (*Ziziphus jujuba* Mill.) reddening [12]. *UGT75C1* gene has an important function in anthocyanin accumulation and coloration in flowers and fruits [33, 34]. In *Lonicera japonica* flowers, the expression levels of *ANS* and *UGT75C1* were upregulated in white and yellow petals compared to green petals, which contributed to the increased accumulation of pelargonidin and cyanidin [35].

Considering that the significant difference of 13 anthocyanin contents was found in four *C. oleifera* petals, we found that the cyanidins synthesis pathway was the most dominant branch in three anthocyanin biosynthesis pathways of *C. oleifera* petals, and five structural genes (*Co4CL1*, *CoF3H1*, *CoANS*, *CoUGT75C1-4*, and *CoUGT75C1-5*) were screened from the differentially expressed genes, which were speculated that those five genes might display essential roles in anthocyanin accumulation. In this study, the transcriptome and quantitative expression results revealed that Co*4CL1* gene exhibited high expression levels in P, CP, and R petals, and was positively regulated the anthocyanins biosynthesis, which was consistent with the results that *Cj4CL* (CSS0016246) showed a significant positive correlation with the total anthocyanins content in the pink and red *C. japonica* petals [6], indicating that Co*4CL1* was the key candidate gene responsible for the anthocyanins accumulation. *CoF3H1* were highly expressed in pink, candy pink, and red petals, compared to that in white petals, which enhanced the transformation of naringenin to dihydrokaemperol, which was in agreement with the results that the *MdF3H* gene significantly led to the high accumulation of anthocyanin in ‘Granny Smith’ apple peel [36]. Moreover, the expression of *CoF3'H* from cyanidin was higher than that of *CoF3′5'H* from delphinidin branch, which was consistent with the metabolite profile analysis that the sum contents of cyanidin and peonidin was much greater than the sum content of delphinidin, which was confirmed again that cyanidin-derivatives were the main coloring substances in *C. oleifera* petals. Therefore, the high expression levels of *CoF3'H* were related to the accumulation of cyanidin-based anthocyanins in *C. oleifera* petals.

*ANS* is the key enzymes involved in anthocyanin biosynthesis, and the overexpression of *SmANS* resulted in the anthocyanins significantly accumulated in *Salvia miltiorrhiza*, and led to the purple-red phenotype [37]. Additionally, an increase in the transcript levels of the *CoF3'H* and *CoANS* genes led to the transformation of leucocyanidin to a colored cyanidin. Two *UFGT* genes encoding for anthocyanidin 3-O-glucosyltransferase were predicted to be responsible for red coloration in *C. reticulata* petals [3]. The cyanidin-3-O-glucoside and pelargonidin-3-O-glucoside contents in pink and candy pink petals were consistent with the expression levels of *UFGT2*. In addition, the high expression level of *CoUGT75C1-4* and *CoUGT75C1-5* genes were related to the anthocyanin biosynthesis in pink, candy pink, and red petals. Therefore, we speculate that the differential expression of *Co4CL1*, *CoF3H1*, *CoANS*, *CoUGT75C1-4*, and *CoUGT75C1-5* were linked to the biosynthesis of cyanidin-3-O-glucoside, cyanidin-3-O-galactoside, cyanidin-3-O-rutinoside, peonidin-3-O-glucoside, cyanidin-3-O-(6''-O-malonyl) glucoside, and cyanidin-3-O-(6''-O-p-Coumaroyl) glucoside, which were the important contributors to the petal color diversity in *C. oleifera* in the present study.

### Transcription factors related to anthocyanin biosynthesis in *C. oleifera* petals

Transcription factors involved in regulating anthocyanin biosynthesis have been identified in plants [38, 39]. Anthocyanin biosynthesis was regulated by a complex network via the co-functioning of multiple structural genes and transcription factors [40, 41]. Anthocyanin biosynthesis was associated with up-regulation of transcription factors in the MYB, bHLH, WRKY, and MADS-box families [42, 43]. MYB TFs have been reported to regulate the anthocyanins biosynthesis and accumulation in *Prunus* peel [44], *Zanthoxylum bungeanum* Maxim. [45], sweet *Osmanthus* fruit [46], apple [47], peach [48], and longan [32]. In addition, the bHLH TFs could interact with MYB and WD40 to form a protein complex, and then regulate anthocyanin biosynthesis [49, 50].

Three MYBs (*CoMYB1*, *CoMYB4*, and *CoMYB44-3*), three bHLHs (*CobHLH30*, *CobHLH77*, and *CobHLH79-1*), and two WRKYs (*CoWRKY7* and *CoWRKY22*) with differential expression were identified in four *C. oleifera* petals based on WGCNA analysis, which were significantly positive with one or more anthocyanins (*P* < 0.05). The WGCNA analysis also suggested that *CoNAC25*, and *CobZIP16-2* had expression levels and were significantly negative with one or more anthocyanins (*P* < 0.05). These transcription factors could directly act on the promoter of anthocyanin structural genes to promote transcription, or regulate the expression of structural genes by binding to other TFs, which finally act on the regulation of anthocyanin biosynthesis [19, 51].

Six structural genes (*Co4CL1*, *CoF3H1*, *CoF3'H*, *CoANS*, *CoUGT75C1-4*, and *CoUGT75C1-5*) had high expression levels and led to the anthocyanin accumulation, and which might be regulated by those transcription factors. Indeed, the promoter regions of *CoF3′H* (Table S8) and *CoANS* (Table S9) all contained MYB and bHLH binding elements. We therefore hypothesized that the candidate transcription factors bHLHs (*CobHLH30*, *CobHLH77*, and *CobHLH79*) and MYBs (*CoMYB1*, *CoMYB4*, *CoMYB20*, and *CoMYB44-3*) might have regulated *CoF3′H* and *CoANS* expression through direct binding to the promoter regions. However, this is the first study to discover the possible regulatory network for anthocyanin biosynthesis in the *C. oleifera* petals. Whether these bHLHs (*CobHLH30*, *CobHLH77*, and *CobHLH79*) interacted with MYBs (*CoMYB1*, *CoMYB4*, *CoMYB20*, and *CoMYB44-3*) to act on the regulation of anthocyanin biosynthesis in *C. oleifera* petals also need to be explored and verified in the future experiments.

## Conclusions

In summary, transcriptome and metabolome analyses of *C. oleifera* petals with different color were performed. The pink, candy pink, and red color formation of *C. oleifera* petals was primarily caused by cyanidin-3-O-glucoside, cyanidin-3-O-galactoside, cyanidin-3-O-rutinoside, peonidin-3-O-glucoside, cyanidin-3-O-(6''-O-malonyl) glucoside, and cyanidin-3-O-(6''-O-p-Coumaroyl) glucoside. Among the differentially expressed structural genes related to the anthocyanin biosynthesis pathway, the high expression of *Co4CL1*, *CoF3H1*, *CoF3'H*, *CoANS*, *CoUGT75C1-4*, and *CoUGT75C1-5* could be related to the accumulation of cyanidin-based and peonidin-based anthocyanins in *C. oleifera* petals. Based on WGCNA analysis, three MYBs (*CoMYB1*, *CoMYB4*, and *CoMYB44-3*), three bHLHs (*CobHLH30*, *CobHLH77*, and *CobHLH79-1*), and two WRKYs (*CoWRKY7* and *CoWRKY22*) could be identified as candidate TFS genes related to anthocyanins biosynthesis, and were contributed to the pink candy pink, and red phenotypes. In conclusion, the results revealed the unique mechanism of color formation in *C. oleifera* petals with different color, and provided valuable insights for further study on the complex molecular network of anthocyanin biosynthesis in *C. oleifera* petals for comprehensive utilization.

### Supplementary Information

**Additional file 1: Fig. S1. **Heatmap of the metabolites in W, P, CP and R petals. **Fig. S2. **The co-expressed genes in W, P, CP,  and R petals. **Table S1.**  Primer sequences used for the qRT-PCR validation.** Table S2. **Flavonoid metabolome profile in Camellia oleifera petals. **Table S3.** Differentially accmulated anthocyainins in W_vs_P comparsion. **Table S4.** Differentially accumulated anthocyanins  in P_vs _CP comparsion. **Table S5.** Differentially accumulated anthocyanins  in CP_vs_R comparsion. **Table S6.** Transcriptome sequencing of C. oleifera petals. **Table S7.** The expression levels of key differentially expressed genes. **Table S8.** Cis-acting elements present in the CoF3′H promoter. **Table S9.** Cis-acting elements present in the CoANS promoter.

## Data Availability

These sequence data have been submitted to the SRA database under accession number PRJNA 859399. All data generated or analyzed during this study are included in this published article and its supplementary information files. The data used to support the findings of this study are available from the corresponding author on reasonable request.
