# Supplementary figures and images for: Integrative metabolome and transcriptome analyses reveal the coloration mechanism in Camellia oleifera petals with different color
Source: BMC Plant Biol. 2024 Jan 2;24:19. doi: 10.1186/s12870-023-04699-6 (PMC10759395; doi:10.1186/s12870-023-04699-6)

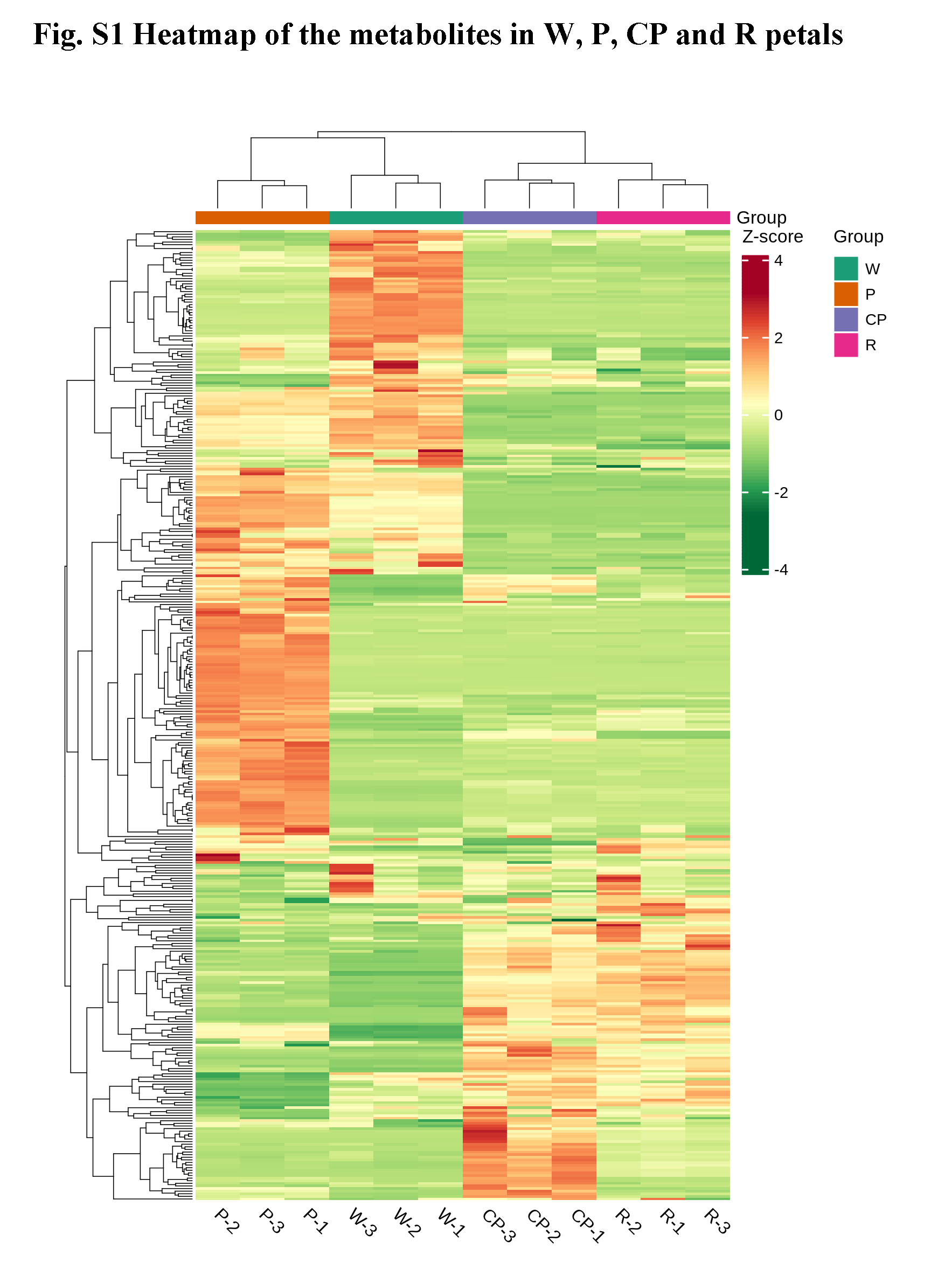

Supplement: Supplementary file 1 — Additional file 1: Fig. S1. Heatmap of the metabolites in W, P, CP and R petals. Fig. S2. The co-expressed genes in W, P, CP, and R petals. Table S1. Primer sequences used for the qRT-PCR validation. Table S2. Flavonoid metabolome profile in Camellia oleifera petals. Table S3. Differentially accmulated anthocyainins in W_vs_P comparsion. Table S4. Differentially accumulated anthocyanins in P_vs _CP comparsion. Table S5. Differentially accumulated anthocyanins in CP_vs_R comparsion. Table S6. Transcriptome sequencing of C. oleifera petals. Table S7. The expression levels of key differentially expressed genes. Table S8. Cis-acting elements present in the CoF3′H promoter. Table S9. Cis-acting elements present in the CoANS promoter. [file 12870_2023_4699_MOESM1_ESM.zip › Supplementary material/Fig. S1.tif]
